# Gross hematuria and IgA nephropathy flare-up following the first dose of Moderna vaccine: A case report

**DOI:** 10.1097/MD.0000000000032524

**Published:** 2022-12-30

**Authors:** Po-Hung Tseng, Shu-Han Chuang, Yueh Pan, Hung-Jen Shih, Chin-Pao Chang, Sheng-Hsien Huang

**Affiliations:** a Division of Urology, Department of Surgery, Changhua Christian Hospital, Changhua, TaiwanROC; b School of Medicine, College of Medicine, Taipei Medical University, Taipei, Taiwan, ROC; c Ph.D. Program in Translational Medicine, National Chung Hsing University, Taichung, Taiwan, ROC; d Rong Hsing Research Center for Translational Medicine, National Chung Hsing University, Taichung, Taiwan, ROC; e Department of Urology, School of Medicine, College of Medicine, Taipei Medical University, Taipei, Taiwan, ROC.

**Keywords:** COVID-19 mRNA vaccine, hematuria, IgA nephropathy, Moderna vaccine

## Abstract

Since the mass vaccination for COVID-19, several case reports indicated the risk of autoimmune disease flare-ups after the vaccination. Among them, COVID-19 vaccine-induced glomerular diseases have drawn attention worldwide. The cases demonstrating the association between the mRNA vaccine and IgA nephropathy (IgAN) exacerbation had been noticed. Mostly mentioned, the flare-ups usually occurred after the second dose.

**Methods:**

We present a Taiwanese female with IgAN who developed gross hematuria within only six hours after the first dose of the Moderna vaccine.

**Results:**

Six hours after the first dose of Moderna vaccine on 8 June 2021, the patient developed gross hematuria and significantly decreased urine output. All symptoms resolved spontaneously on the fifth day after the vaccination without any intervention. On the fourth day after the vaccination, the patient were able to back to her original condition.

**Conclusion:**

This was an intriguing case of IgAN flare-up following the first dose of mRNA-based COVID-19 vaccination.

## 1. Introduction

The COVID-19 vaccine-induced glomerular disease have drawn attention worldwide.^[1]^ Numerous international reports demonstrated coronavirus disease 2019 (COVID-19) vaccines, especially for messenger ribonucleic acid (mRNA) vaccines, might trigger unexpected autoimmune responses^[2]^ and even the sudden onset of glomerular diseases.^[3,4]^ However, among the previously reported cases collected by Bomback et al,^[5]^ the IgA nephropathy (IgAN) exacerbation usually occurred after the second dose and only 2 cases, which were reported by Perrin et al,^[6]^ occurred after the first dose. In this case report, we have a Taiwanese female with IgAN who developed gross hematuria within only 6 hours after the first dose of the Moderna vaccine.

## 2. Case history

A 40-year-old Taiwanese female with IgAN was proven by renal biopsy ten years ago. She has been under stable condition with medication of oral steroids and angiotensin II receptor antagonists ever since. She denied hypertension or hematuria within 3 months. She only stopped taking steroids for 7 days before the administration of the Moderna vaccine. 6 hours after the first dose of Moderna vaccine on June 8, 2021, she developed gross hematuria and significantly decreased urine output. She also reported nonspecific side effects including mild febrile (37.8°C), headache, stiff neck, and fatigue. All symptoms resolved spontaneously on the fifth day after the vaccination without any intervention.

Thirty-eight days after the first dose, the patient received her second dose of the Moderna vaccine. This time, her symptoms of gross hematuria and fever episode over 38.6°C occurred 1 day later. The fever persisted only for 1 day and the gross hematuria accompanied by decreased urine output lasted for 4 days. She came to our outpatient department and followed up on her laboratory data of urine test on the same day of the second dose and 1 day after the development of gross hematuria, respectively. Hematuria deteriorated with an increase in urine red blood cells by a random urine test. On the fourth day after the vaccination, she had already been back to her original condition.

## 3. Discussion

With mass vaccination for COVID-19, several case reports put the risk of autoimmune disease flare-ups after the vaccination in the spotlight.^[2,5]^ Exacerbation of immune-mediated glomerular diseases following the COVID-19 vaccine, especially for the mRNA-based vaccine (either Pfizer-BioNTech or Moderna) has been thoroughly reported by Bomback et al,^[5]^ with the collection of 6 reports including 10 cases of IgA nephropathy presenting as gross hematuria following the mRNA-based vaccine. Among these patients, 6 patients received Moderna vaccination while the other 4 received Pfizer. Most of these cases occurred gross hematuria within 2 days after the second dose. However, there were 2 cases reported by Perrin et al,^[6]^ in which one received Pfizer and the other take Moderna, to develop gross hematuria soon after the first dose.

IgAN is the most common glomerulonephritis caused by mesangial deposition of autoantibodies of IgA.^[7]^ Previous studies have shown the potential link between influenza vaccination with flare-up of IgA nephropathy.^[8]^ Cases of reactivation of IgA vasculitis occurring after COVID-19 vaccination had also been reported.^[9]^ Furthermore, Rahim et al^[4]^ reported a recurrence of IgA nephropathy after COVID-19 vaccination in the same patient who had a similar episode 2 years earlier after receiving a recombinant zoster vaccine (Shingrix).

All above studies indicated vaccine-induced IgA response is not uncommon and could take place at any vulnerable targets. To the best of our knowledge, severe COVID-19 illnesses can trigger an IgA response in the bronchial mucosa.^[10]^ Taken together, these results might suggest a link between the virus antigen-containing COVID-19 vaccine and an increase in anti-SARS-CoV-2 spike IgA. While coincidence cannot be excluded, the close temporal association within days between vaccination may suggest a potential pathogenetic linkage.

There are several debates concerning the link between autoimmune disease flares after COVID-19 vaccination,^[5]^ but still, the mechanism remains unclear. Whether activation of autoreactive B cells after COVID-19 vaccination causes the preexisting or de novo mobilization of autoreactive B cells producing IgA (or both) remains to be established. According to the studies by Bomback et al,^[5]^ T cells are assumed to be the key mediators of COVID-19 vaccine-induced glomerular disease because these responses are similar to the infection of SARS-Cov-2 itself to cause activation of diverse autoimmune and alloimmune diseases affecting the kidney. Kudose et al^[3]^ also assumed it is a systemic cytokine-mediated flare, possibly via induction of heightened IgA1 antiglycan immune responses. Rahim et al^[4]^ suggested it is related to the delayed-type hypersensitivity owing to the response usually occurring after the second dose. But it became doubtful due to more and more reports of events occurring within 24 hours after the first dose, including 2 cases reported by Perrin et al^[6]^ and our case. Only one thing in common is all these cases highlight that in the absence of intervention, COVID-19 vaccine-associated IgAN and immunoglobulin A–associated vasculitis flairs may improve spontaneously.

Without a doubt, these reports should not lead to vaccine hesitation as the benefits of vaccination strongly outweigh the potential risks. But they bring caution on patients with IgAN should be monitored closely following the COVID-19 vaccine. If the adverse events after the vaccination could not resolve, shifting to another vaccine should be taken into consideration. Further discussion of how to incorporate active glomerular disease after the COVID-19 vaccination into a current therapeutic plan for these patients is an issue.

## 4. Conclusion

To sum up, we present an intriguing case of IgAN flare-up following the first dose of mRNA-based COVID-19 vaccination. The optimal therapy of COVID-19 vaccine-associated glomerular diseases including IgAN remains unclear. Our patient’s disease has been in remission with normal renal function and blood pressure after a complete 2-dose of vaccination. Nevertheless, further studies are required to investigate the exact pathogenesis of COVID-19 vaccine-induced IgAN. Is it necessary to shift to a different type of vaccine, such as traditional inactivated viral and adjuvanted protein vaccines, after the adverse events of the first dose? Is it possible for those adverse events processing speed up the deterioration of a patient’s renal function eventually? How to incorporate active glomerular disease after the COVID-19 vaccination into a current therapeutic plan for these patients has become an important issue.

## Author contributions

**Conceptualization:** Po-Hung Tseng, Shu-Han Chuang, Hung-Jen Shih, Sheng-Hsien Huang.

**Data curation:** Po-Hung Tseng, Shu-Han Chuang.

**Formal analysis:** Po-Hung Tseng.

**Funding acquisition:** Hung-Jen Shih.

**Investigation:** Po-Hung Tseng, Shu-Han Chuang.

**Methodology:** Po-Hung Tseng, Shu-Han Chuang, Hung-Jen Shih, Hung-Jen Shih, Chin-Pao Chang.

**Project administration:** Shu-Han Chuang.

**Resources:** Yueh Pan, Hung-Jen Shih, Sheng-Hsien Huang.

**Software:** Hung-Jen Shih.

**Supervision:** Yueh Pan, Hung-Jen Shih, Chin-Pao Chang, Sheng-Hsien Huang.

**Validation:** Po-Hung Tseng, Yueh Pan, Hung-Jen Shih.

**Visualization:** Po-Hung Tseng, Shu-Han Chuang.

**Writing—original draft:** Po-Hung Tseng, Shu-Han Chuang.

**Writing—review and editing:** Po-Hung Tseng, Shu-Han Chuang, Yueh Pan, Hung-Jen Shih, Chin-Pao Chang, Sheng-Hsien Huang.

## References

[1] Caza TN Cassol CA Messias N. Glomerular disease in temporal association with SARS-CoV-2 vaccination: a series of 29 cases. Kidney360. 2021;2:1770–80. 35372991 10.34067/KID.0005372021 PMC8785835

[2] Ishay Y Kenig A Tsemach-Toren T. Autoimmune phenomena following SARS-CoV-2 vaccination. Int Immunopharmacol. 2021;99:107970. 34280851 10.1016/j.intimp.2021.107970 PMC8270741

[3] Kudose S Friedmann P Albajrami O. Histologic correlates of gross hematuria following moderna COVID-19 vaccine in patients with IgA nephropathy. Kidney Int. 2021;100:468–9. 34146600 10.1016/j.kint.2021.06.011 PMC8214418

[4] Rahim SEG Lin JT Wang JC. A case of gross hematuria and IgA nephropathy flare-up following SARS-CoV-2 vaccination. Kidney Int. 2021;100:238. 10.1016/j.kint.2021.04.024 PMC8079938 33932458

[5] Bomback AS Kudose S D’Agati VD. De novo and relapsing glomerular diseases after COVID-19 vaccination: what do we know so far? Am J Kidney Dis. 2021;78:477–80. 34182049 10.1053/j.ajkd.2021.06.004 PMC8230841

[6] Perrin P Bassand X Benotmane I. Gross hematuria following SARS-CoV-2 vaccination in patients with IgA nephropathy. Kidney Int. 2021;100:466–8. 34087252 10.1016/j.kint.2021.05.022 PMC8166778

[7] Ebefors K Liu P Lassén E. Mesangial cells from patients with IgA nephropathy have increased susceptibility to galactose-deficient IgA1. BMC Nephrol. 2016;17:40. 27044423 10.1186/s12882-016-0251-5 PMC4820936

[8] Kavukçu S Soylu A Sarioğlu S. IgA nephropathy in mice following repeated administration of conjugated haemophilus influenzae type B vaccine (PRP-T). Tokai J Exp Clin Med. 1997;22:167–74. 9777007

[9] Obeid M Fenwick C Pantaleo G. Reactivation of IgA vasculitis after COVID-19 vaccination. Lancet Rheumatol. 2021;3:e617. 34250509 10.1016/S2665-9913(21)00211-3 PMC8260100

[10] Hasan Ali O Bomze D Risch L. Severe coronavirus disease 2019 (COVID-19) is associated with elevated serum immunoglobulin (Ig) A and antiphospholipid IgA antibodies. Clin Infect Dis. 2021;73:e2869–74. 32997739 10.1093/cid/ciaa1496 PMC7543315

